# Airport/seaport and autochthonous malaria in Europe from 1969 to 2022: A systematic review

**DOI:** 10.1016/j.nmni.2025.101627

**Published:** 2025-08-24

**Authors:** Daniela Balzli, Nejla Gültekin, Zeno Stanga, Ismail Ülgür, Nadja Hedrich, Jan Fehr, Patricia Schlagenhauf

**Affiliations:** a University of Zurich, Department of Public and Global Health, Institute for Epidemiology, Biostatistics and Prevention, Hirschengraben 84, 8001, Zurich, Switzerland; b Centre of Competence for Military and Disaster Medicine, Swiss Armed Forces, Bern, Switzerland; c University of Zurich Centre for Travel Medicine, WHO Collaborating Centre for Travellers' Health, Department of Public and Global Health, MilMedBiol Competence Centre, Institute for Epidemiology, Biostatistics and Prevention, University of Zurich, Hirschengraben 84, 8001, Zurich, Switzerland

**Keywords:** Airport, Seaport, Autochthonous, Malaria, Europe

## Abstract

**Background:**

In the context of this paper, airport/seaport malaria denotes the accidental relocation by air or sea of a malaria infected mosquito to Europe, a non-endemic area, the survival of the transported mosquito and subsequent blood meal and infection of a local person. Autochthonous malaria refers to locally transmitted cases of malaria in Europe.

**Methods:**

The systematic review followed PRISMA guidelines and was registered on PROSPERO (CRD42023444243). PubMed and Ovid MEDLINE electronic databases as well as EMBASE, Scopus and CINAHL were searched for eligible papers. The selection process followed strict inclusion and exclusion criteria.

**Results:**

We included 68 papers describing 115 cases of airport, seaport or autochthonous malaria in Europe, with a total of 68 airport/seaport malaria cases and 47 autochthonous malaria cases. France, Germany, Italy and Spain reported both types of malaria cases. Cases of airport/seaport malaria only were reported from Belgium, Luxembourg, Switzerland and the United Kingdom. Cases of autochthonous malaria only were reported from Greece, Malta and the Netherlands. The case fatality rates for airport/seaport malaria were 13.24 % and for autochthonous malaria 2.13 % respectively.

**Conclusion:**

The importance of airport/seaport and autochthonous malaria is related to the frequently delayed or missed diagnosis, leading to high case fatality rates. Rising temperature may facilitate the importation and proliferation of competent *Anopheles* vectors. Increased human migration and travel with malaria parasite carriage may contribute to the reemergence of autochthonous malaria in Europe.

## 1 Introduction

Malaria is a vector-borne disease, caused by *Plasmodia* parasites, which are transmitted through the bite of infected female *Anopheles* mosquitoes. Infections in humans can be caused by five parasite species, but *P. falciparum* and *P. vivax* have the highest transmission numbers [1]. There are around 430 known *Anopheles’* species, of which 30–40 species are capable of transmitting *Plasmodia* parasites and of these some 16 European *Anopheles* species have potential to transmit malaria [2]. Anopheles mosquitos are mostly found in tropical and subtropical areas, as they prefer a warm, wet and moist climate. Endemic malaria regions are located mainly in Sub-Saharan Africa, Asia and South America.

In Europe malaria was endemic until the 20th century. With drainage of swamps and marshy areas, and the application of insecticides malaria could be eliminated. For example, France is considered malaria free since 1943, Corsica since 1960 [3], Greece since 1974 [4], Germany since the 1950s [2] and Spain since 1964 [5]. The eradication in Europe was successful in the 1970s [6]. Currently Europe is considered non-endemic for malaria infection.

According to the WHO World Malaria Report 2022 [7], the WHO European region is malaria free since 2015 with the last reported indigenous malaria case in Tajikistan, which occurred in 2014. In comparison to endemic areas, where an estimated total of 247 million malaria cases are registered, of which 629′000 had a fatal outcome. This is an increase of 2 million cases and a decrease of 6′000 fatal cases compared to the 2020.

Despite its “non-endemic” status it is possible to acquire a malaria infection without leaving Europe, without having travelled to a malaria endemic region.

“*Autochthonous*” is a term which is often used in relation to infectious diseases. An autochthonous transmission or infection means that the acquisition of an infectious disease occurs locally in a non-endemic area and is not imported from travel abroad.

Apart from autochthonous transmission, there are other types of local malaria transmission including airport, baggage and seaport malaria, runway malaria, nosocomial transmission as occurs with blood transfusions or organ transplantation.

The airport and baggage form includes malaria infection acquired after a bite of an infected *Anopheles* mosquito, which was transported by airplane from an endemic region to a non-endemic region. Mosquitoes can be transported in the cabin, as well as in the cargo space. A first case was reported in 1969 [8]. Without a suspicion of travel to malaria endemic areas, health care professionals mostly exclude the possibility of the diagnosis of a malaria infection. However, our systematic review focused on malaria acquired in Europe and the details of these cases, in which countries they occurred, the presentation and the circumstances behind each occurrence.

This systematic review reviews reports of autochthonous and airport/seaport forms of malaria reported in the literature.

## 2 Methods

The systematic review was conducted according to the Preferred Reporting Items for Systematic Reviews and Meta-Analyses (PRISMA) guidelines [9]. A prior registration of the protocol for this systematic review was done on PROSPERO (CRD42023444243) [10].

Using the PICO format, our research question is divided in the following sections: 1) Patients with the diagnosis of non-imported malaria. 2) The transmission of infection was classified as airport/seaport or autochthonous. 3) A comparison of the described results of airport versus autochthonous malaria cases. 4) Outcome describes as recovery or death.

This led us to the following research question: What is the current data situation regarding publications on airport and autochthonous malaria since the first case description in 1969?

According to this background, the selection process was based on database searches using keywords that have been predetermined by two main authors (DB, PS). Those keywords included “autochthonous malaria; airport malaria; Europe”. Publications in English, German and French published up to February 2024, were included. The cases must have occurred in between 1969 and 2022.

We used the PubMed and Ovid MEDLINE electronic databases and EMBASE, Scopus and CINAHL for our literature search. This systematic search process was done by the primary study researcher DB in collaboration with a librarian scientist. The search history was documented on an excel sheet.

The results, titles and abstracts of these searches were checked for duplications and all identical publications were removed. As a next step the remaining list of publications was scrutinized by two researchers (DB, PS) according to inclusion and exclusion criteria shown in Table 1. Those publications that satisfy our search criteria were further considered and the process is shown in Prisma flow diagram (Table 2). Any discrepancies were discussed with a third researcher and resolved at any step.

**Table 1:** Inclusion and exclusion criteria for the Systematic Review: **Airport/seaport and autochthonous malaria in Europe from 1969 to 2022**.

Inclusion criteria	Exclusion criteria
Cases occurring in Europe - defined as the European Union of 27 countries plus the three additional EEA countries Iceland, Liechtenstein and Norway, plus the United Kingdom and Switzerland	nosocomial transmission, congenital cases, transfusion of blood (-products), organ transplantation and travel-related cases
Confirmed non-imported malaria infection	Cases infected in the European Union overseas countries and territories (Aruba, Bonaire, Curaçao, French Polynesia, French Southern and Antarctic Territories, Greenland, New Caledonia, Saba, Saint Barthélemy, St. Eustatius, Sint Maarten, St. Pierre and Miquelon, Wallis and Futuna), European Union outermost regions (French Guiana, Guadeloupe, Martinique, Mayotte, Reunion Island and Saint-Martin, Azores and Madeira, The Canary Islands) or British Overseas Territories (Anguilla, Bermuda, British Antarctic Territory, British Indian Ocean Territory, British Virgin Islands, Cayman Islands, Dhekelia, Falkland Islands, Gibraltar, Montserrat, Pitcairn, Henderson, Ducie and Oeno Islands, St. Helena, Ascension and Tristan de Cunha, South Georgia and South Sandwich Islands, Turks and Caicos Islands).
All age, ethnicity and gender	
All type of studies	
Case occurrence in the years from 1969 to 2022

**Table 2:**
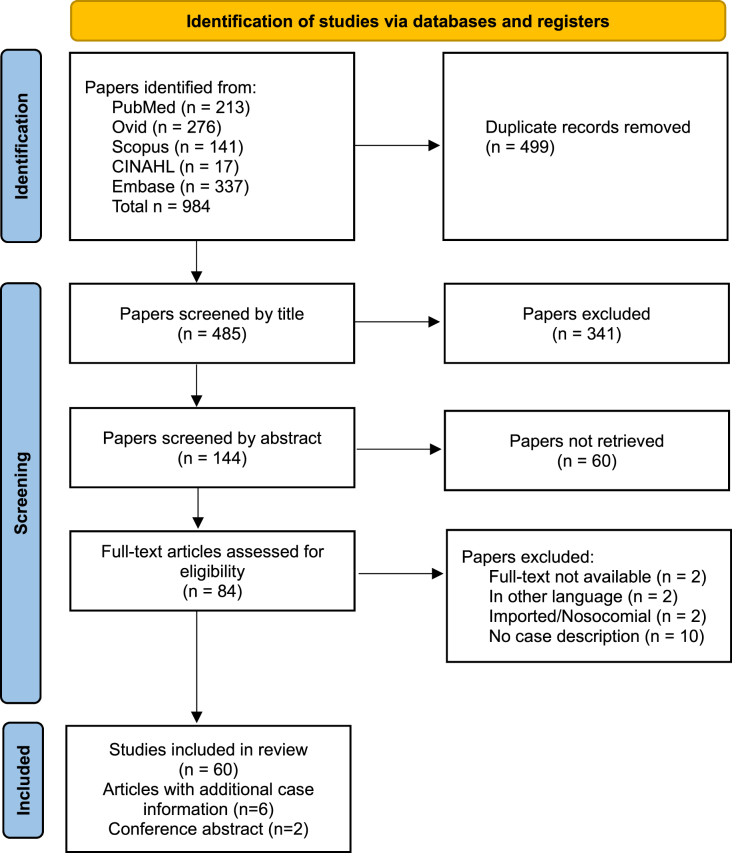
*PRISMA flow diagram*. **Airport/seaport and autochthonous malaria in Europe from 1969 to 2022** [9]

The publications that were selected had then been added to the Rayyan website and checked again by both researchers before relevant extraction.

Publications containing no case descriptions, but giving some background information, were taken into account for the discussion.

The data which was extracted in an Excel file included the following: age, gender, country of origin of the case, citizenship, occupation (e.g. airport worker), history of travel (if any), clinical data, malaria diagnostic data, time to presentation, treatment, outcome, genomic analysis (if available), time of the year of case occurrence, proximity to airport, local “*Anopheles*” vector information and further considerations (if there were other possible explanations). Our approach was a narrative synthesis of all the available data. We then used descriptive statistics to describe the epidemiology, case occurrence and outcome.

## 3 Results

We identified 984 papers in the electronic database search and after removal of all duplicates there were 485 papers for screening. The screening process of first the titles and then the abstracts left 84 papers for full-text screening. Out of those 68 articles matched the inclusion criteria – 37 papers for airport/seaport malaria and 29 papers plus 2 conference abstracts for autochthonous malaria.

An overview regarding the number of papers and details of the cases sorted by country, number of deaths and recovery, sex and type of malaria, and split according to airport/seaport and autochthonous classification, are shown in Table 3. (see Table 3a, Table 3ba, b).

**Table 3:** Overview of malaria cases acquired in Europe according to country, outcome, sex and species of malaria.

	Country	Papers	Cases	Recovery	Death	N/A	male	Female	N/A	*P. falcipa-rum*	*P. ovale*	*P. malariae*	*P. vivax*	Mixed
**Airport malaria**	Germany	5	7	7			3	1	3	7				
	United Kingdom	1	2	2			1	1		2				
	Italy	3	3	2		1		2	1	3				
	Spain	2	2	1	1			2		1	1			
	Belgium	6	17	12	4	1	13	4		16	1			
	France	12	23	15	3	5	18	5		19			1	3
	Luxem-bourg	1	5			5	1	4		5				
	Switzer- land	4	6	5	1		5	1		6				

**Seaport**	France	2	2	2			1	1		2				
	Belgium	1	1	1				1		1				

**Autochtho-nous**	Greece	7	15	12		3	9	6					15	
	Germany	3	5	4		1	2	3		5			4	
	Spain	5	5	4		1	3	1	1	1				
	Italy	6	10	6	1	3	6	4		4	2	1	3	
	France	8	9	9			8	1		6		1	2	
	Netherlands	1	2	2			1	1		2				
	Malta	1	1	1			1							

**Total**		68	115	85	10	20	72	38	5	81	4	2	25	3

**Table 3a:** Specification of Airport malaria cases with first author, country and year of case occurrence.

Airport Malaria			
First author	Country	Year of case occurrence	Reference
F. Praetorius	Germany	1997	(11)
F. Praetorius	Germany	1997	(12)
I. Rabinowitz	Germany	Published 2004	(13)
I. Wieters	Germany	2019	(14)
J. Kessel	Germany	2022	(15)

D. Whitfield	United Kingdom	1983	(16)

M. A. Rosci	Italy	1985	(17)
F. Rizzo	Italy	Published 1989	(18)
F. Castelli	Italy	1992	(19)

J.I. Alos	Spain	1984	(20)
J. Cuadros	Spain	2001	(21)

G. Holvoet	Belgium	1982	(22)
WHO	Belgium	1983	(23)
WHO	Belgium	1986	(24)
J. Van den Lynen	Belgium	1995	(25)
C. Theunissen	Belgium	2008	(26)
W. Van Bortel	Belgium	2020	(27)

J. M. Doby	France	1969	(8)
T. Giacomini	France	1976 + 1977	(28)
A. Larcan	France	1977	(29)
P. Saliou	France	1978	(30)
T. Giacomini	France	1994	(31)
T. Giacomini	France	1994	(32)
F. Poupin	France	1994	(33)
M.T. Baixench	France	1995	(34)
D. Lusina	France	1999	(35)
P. Marty	France	2000	(36)
C. Pomares-Estran	France	2008	(37)
S. Gallien	France	2013	(38)

R. Hemmer	Luxembourg	1997 + 1999	(39)

M. Bouvier	Switzerland	1989	(40)
G. Majori	Switzerland	1989	(41)
WHO	Switzerland	1990	(42)
WHO	Switzerland	1996	(43)

J. Delmont	France	1993	(44)
J. Delmont	France	1993	(45)

R. Peleman	Belgium	1997	(46)

**Table 3b:** Specification of Autochthonous malaria cases with first author, country and year of case occurrence.

Autochthonous Malaria			
First author	Country	Year of case occurrence	Reference
P. Andriopoulos	Greece	2009 + 2010	(47)
P. Andriopoulos	Greece	2009	(48)
C. V. Loupa	Greece	2009	(49)
P. Korovessi	Greece	2010	(50)
A. Gougoutsi	Greece	2011	(51)
S. A. Florescu	Greece	2011	(52)
K. Danis	Greece	2011	(4)
A. Ioannidis	Greece	2012	(53)
D. Dimopoulou	Greece	Published 2016	(54)

C. F. Mantel	Germany	1994	(55)
A. Krüger	Germany	1997	(56)
T. Zoller	Germany	2007	(57)

P. Santa-Olalla Peralta	Spain	2010	(58)
J. Lucientes	Spain	2010	(59)
J. M. Rubio	Spain	2011	(60)
L. Barrado	Spain	2014	(61)
B. Verona Mesia	Spain	2022	(62)
E. Velasco	Spain	Published 2017	(5)

M. Sartori	Italy	1986	(63)
M. Baldari	Italy	1997	(64)
R. Romi	Italy	2009 + 2011	(65)
D. Boccolini	Italy	2017	(66)
G. Angeloni	Italy	2017	(67)
L. Zammarchi	Italy	2018	(68)

M. Gentilini	France	1974	(69)
M. Bentata-Pessayre	France	1976	(70)
P. Saliou	France	1977	(71)
J. Andrieu	France	1978	(72)
P. Bégué	France	1983	(73)
P. Marty	France	1991	(74)
B. Doudier	France	2006	(75)
A. Armengaud	France	2006	(76)

J. E. Arends	Netherlands	2012	(77)

R. Medialdea	Malta	2018	(78)

### 3.1 Airport and seaport malaria

After a final screening, we included 31 papers describing 65 cases of airport and 3 cases of seaport malaria between 1969 and 2022. An additional 6 papers described same cases and were used for complementation of previously retrieved information.

The airports involved were Brussels International Airport (Belgium), Roissy-Charles-de-Gaulle and Toulouse-Blagnac International Airport (France), Munich Airport and Frankfurt am Main (Germany), Gatwick Airport (UK), Milan International Airport (Italy), Airport of Luxembourg, Madrid International Airport (Spain) and Geneva International Airport (Switzerland).

Seaports involved were the harbour of Ghent (Belgium) and Marseille (France).

**Fig. 1:**
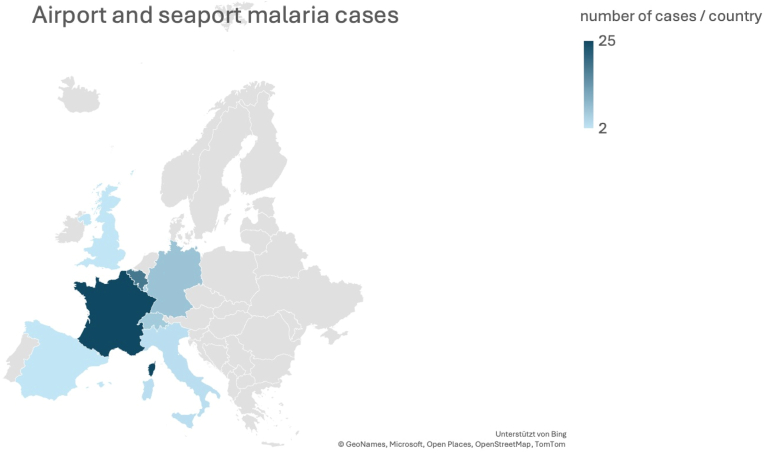
Distribution of airport and seaport malaria cases in Europe.

The distribution based on frequency is shown in Fig. 1 and with airport/seaport specification in Table 4.

**Table 4:** Distribution of airport and seaport malaria cases in Europe.

Country	1969–1990	1990–2010	2010–2022	Airport/Seaport	Total
Germany		2	5	Munich Airport: 1	7
				Frankfurt am Main: 6	
United Kingdom	2			Gatwick: 2	2
Italy	2	1		Ciampino: 1	3
Spain	1	1		Madrid: 1	2
Belgium	8	7	2	Brussel: 16	18
		1		Port of Ghent: 1	
France	6	16	1	Roissy-CdG: 16	25
				Le Bourget: 2	
				Toulouse: 2	
				Nice: 1	
		2		Port de Marseille: 2	
Luxembourg		5		Luxembourg: 5	5
Switzerland	5	1		Geneva: 6	6

**Fig. 2:**
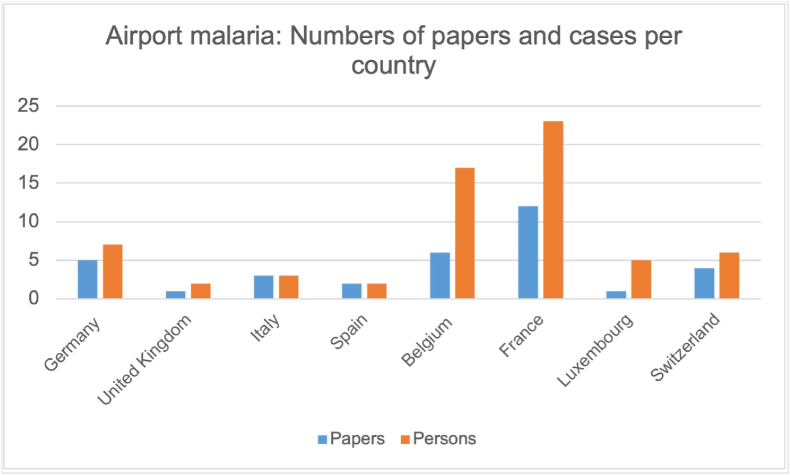
Number of papers and cases for airport malaria.

**Fig. 3:**
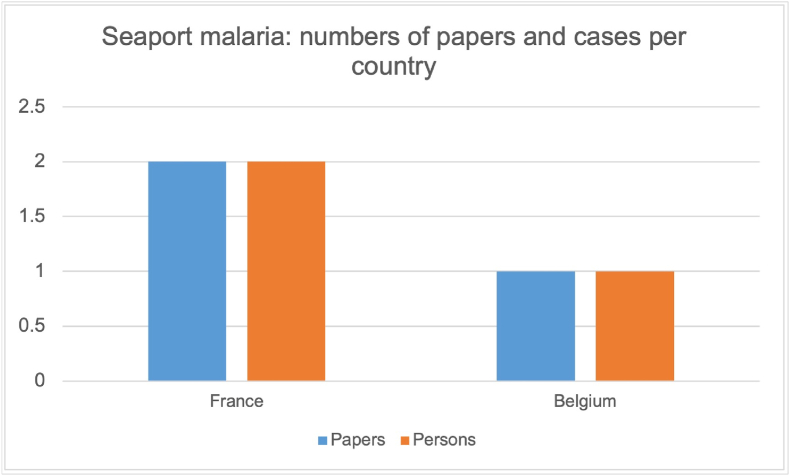
Number of papers and cases for seaport malaria.

The exact number of papers included and persons with malaria acquired in Europe is shown in Fig. 2, Fig. 3.

**Fig. 4:**
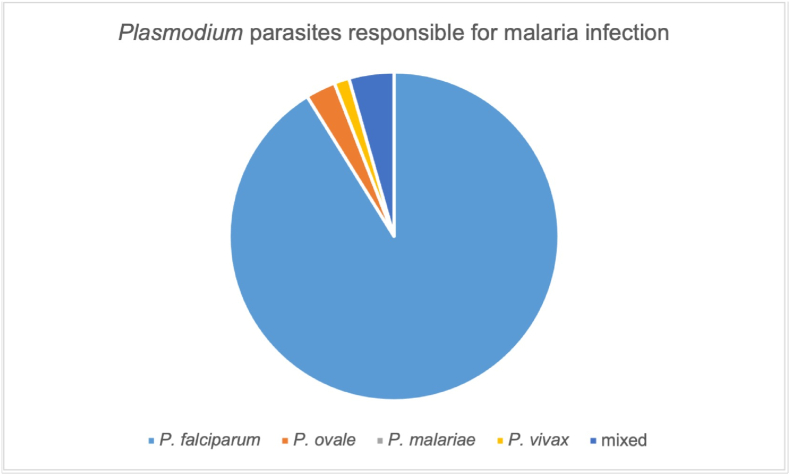
Proportion of Plasmodium spp. in airport/seaport malaria cases in Europe.

*Plasmodium falciparum* was responsible for almost all the infections (62 out of 68), in two cases malaria was due to *Plasmodium ovale* parasites, one case with *Plasmodium vivax* and three cases presented with mixed infections (1x *P. falciparum + P. malariae,* 2x *P. falciparum + P. vivax*). Fig. 4 shows the proportional distribution.

59 out of those 68 persons made a full recovery or no outcome was described. The other 9 patients had a fatal outcome (case fatality rate 13.24 %) with diagnosis only made post-mortem in 2 cases.

50 cases occurred during summer, 9 cases were reported in winter, 4 cases during fall and in the missing 5 cases no timeline was described.

The delay in diagnosis lay between 2 days and 32 days, with a mean value of 10 (10.40) days.

The most common symptoms described include fever, sweating/chills, headache, cough, gastrointestinal complaints (diarrhea and/or vomiting, abdominal pain) and muscle pain. In severe cases patients also presented with neurological symptoms including dizziness, loss of consciousness, seizures or presented in a comatose state.

The above listed unspecific symptoms led often to a misdiagnosis at the first medical consultation. For example cases were diagnosed and treated for pneumonia [20], rhinopharyngitis [31], gastroenteritis [79], cholecystitis [11], pyelonephritis [21,40] or sepsis of unknown origin [11]. Worsening of symptoms with hospital admission and a repeated examination or further investigations due to the onset of new symptoms led to the correct diagnosis of malaria.

In the clinical examination there was a range from normal findings to fever, hepatomegaly with or without splenomegaly, and in some cases jaundice, oliguria, as well as confusion. Nuchal rigidity and signs of encephalopathy were reported in a case with cerebral manifestations.

The most common laboratory findings which were mentioned in the case descriptions were anemia, thrombocytopenia, elevated liver enzymes, signs of hemolysis (elevated bilirubin and LDH), elevated inflammatory markers and less often leukopenia, hyponatremia and signs of coagulopathy.

Complications that have arisen in severe malaria manifestations include respiratory, liver and/or kidney failure [15,27,29] as well as disseminated intravascular coagulopathy with the need for transfer to an intensive care unit, mechanical ventilation [25,31], replacement of red blood cells, platelets or coagulation factors [11,18,20].

Before the correct diagnosis was made, treatment was often initiated with antibiotics, according to the clinical and laboratory findings transfusion of blood products and supportive therapy (mechanical ventilation, renal replacement).

As soon as the diagnosis was made, antimalarial therapy was started as oral or intravenous administration. The range of treatments included mefloquine, quinine±doxycycline or erythromycin, artemisinin, artesunate, atovaquone/proguanil, chloroquine, primaquine, halofantrine, metakelfin (sulphadoxine-pyrimethamine).

Side effects of antimalarial drug therapy were described in 3 cases: Due to vomiting and confusion, which may have been provoked or enhanced by the antimalarial therapy, mefloquine had to be replaced by intravenous quinine in combination with doxycycline in a 60-year-old security worker [25].

After 2 days of treatment with Nivaquine (chloroquine) there was a development of tremor, sweating and swallow paralysis, which was considered as a reaction to the medication. Nevertheless, the therapy was continued for another 8 days in reduced dosage and the police sergeant made a full recovery [80].

A 44-year-old runway maintenance worker was treated with intravenous quinine for 3 days, when he started presenting signs of intolerance with tinnitus and vomiting. The treatment was changed to chloroquine after resistance testing.

Despite adequate treatment and negative parasite study, there was also a relapse after 19 days. He was then treated with halofantrine [[31], [32], [33]].

Two other relapses were mentioned in the case descriptions: One recrudescence occurred with a bus driver in his fifties, 3 days after treatment with artemether and lumefantrine 80/480 mg perorally (second dose after 8 h, then every 12h for total 60 h). This despite the parasite clearance after initial treatment was confirmed by fluorescent microscopy. The relapse was then treated with atovaquone-proguanil for 3 days with all further testing negative [15].

The other relapse occurred around 19 days after the treatment with halofantrine (given 2 times 1 week apart), which resulted in re-hospitalization for fever. This 51-year-old patient was treated first with intravenous quinine and afterwards peroral in combination with erythromycin, each for 3 days [31].

With regard to the occupations of the malaria patients: 24 out of the reported cases were working at the airport. Their jobs included aircraft maintenance (2x Germany), baggage handler (1x Belgium, 2x France), customs officer (5x Belgium), security/police agent (3x Belgium, 2x France, 1x Germany), runway maintenance worker (2x France), parcel delivery truck driver with frequent stops at the airport (1x France), bus driver at the airport (1x Germany), forklift driver (1x Germany), cable worker (1x France), saxophone player of the “Guard républicaine” with musical reception at airports (1x France) or administration at the airport (former pilot - 1x Switzerland).

With regard to the seaport cases, one of the 4 seaport malaria patients worked occasionally as a laborer at the harbor docks in Marseille.

Other malaria cases reported are living close to an airport with their place of residence located within a radius of less than 2 km and up to 100 km away from an airport. In case of a radius outside the flying distance of an *Anopheles* mosquito, the most likely explanation was a transport by luggage or a vehicle coming from the airport [11,16,19,26].

The medical history of 5 people revealed an airport visit (Germany 1x, Belgium 3x, United Kingdom 1x). In 4 cases recent travel by plane to a non-endemic country (Luxembourg 4x) and in 1 case a visit from a friend from Guinea-Conakry (Belgium).

In 17 cases, no work or residence near an airport was described.

### 3.2 Autochthonous malaria

In this sub-group of malaria cases we included 31 papers describing 47 cases of suspected autochthonous cases in the years from 1986 to 2022.

The countries which reported autochthonous cases are France, Greece, Germany, Italy, Malta, the Netherlands and Spain.

**Fig. 5:**
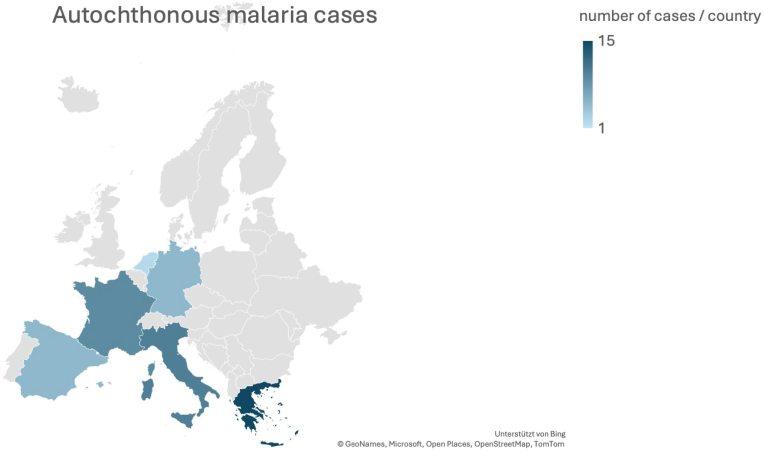
Distribution of autochthonous malaria cases in Europe.

Fig. 5 shows the distribution throughout the European countries based on the reported number of autochthonous cases.

**Fig. 6:**
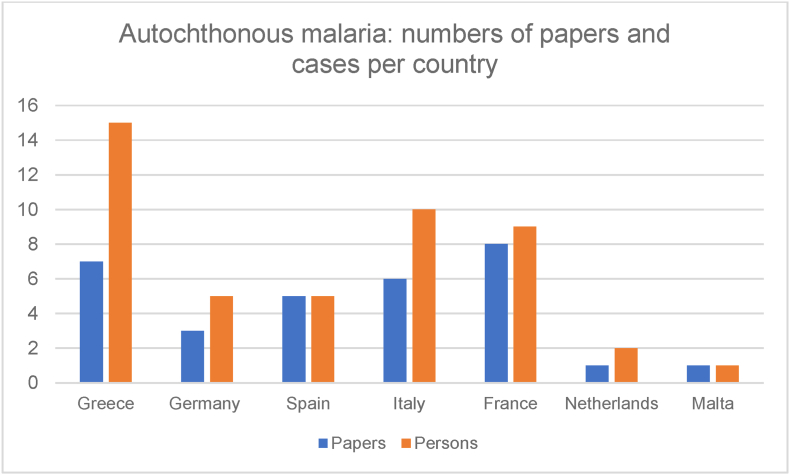
Number of papers and cases for autochthonous malaria.

The exact number of papers and persons affected is shown in Fig. 6.

**Fig. 7:**
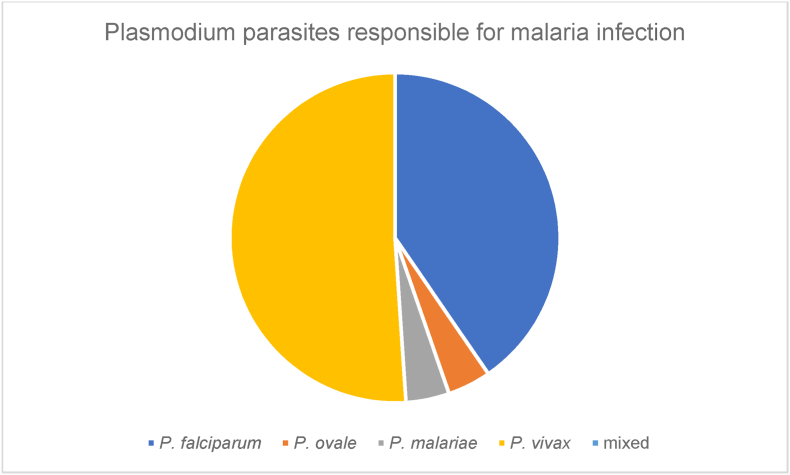
Proportion of Plasmodium spp. in autochthonous malaria cases.

Microscopic and/or genomic analysis showed 19 cases of *P. falciparum* malaria, 24 cases of *P. vivax*, 2 cases of *P. ovale* and 2 cases of *P. malariae* malaria as shown in Fig. 7.

46 out of those 47 persons made a full recovery or no outcome was described. Only one patient had a fatal outcome (case fatality rate 2.13 %).

In 31 cases symptoms started during summertime, 2 cases presented with symptoms during springtime, 9 cases during fall and only 1 case during winter.

The diagnostic delay lay between 1 day and 31 days, with a mean value of 10 (9.88) days. In two cases the diagnosis was only made post-mortem.

The most common presenting symptoms were similar to those of airport malaria manifestation, such as fever with or without shivering/sweating, headache, coughing, general malaise, abdominal complaints (pain, diarrhea, vomiting).

Misdiagnosis made by general practitioners or hospital physicians include tonsillitis [58], pneumonia [5], acute respiratory distress syndrome [51], suspicion of hematological disease [66] or acute nephritis [55].

The clinical examination ranged from absence of any pathologies to reduced general condition, hepatomegaly, splenomegaly, skin pallor or icterus and in individual cases confusion, bilateral abnormal breath sounds in auscultation, hypotension or signs of septic shock.

In two cases physicians did a laparoscopy and saw a blackish liver which was due to the malaria pigment [70,72]. Laboratory findings in the case descriptions were anemia, thrombocytopenia, hyperbilirubinemia, elevated liver enzymes, elevated inflammation markers (CRP), hematuria, negative serological investigation (bacterial and viral tests).

Complications that occurred during the hospital stay were severe coagulopathy, oliguria [55], multiple organ failure with ICU admission [62].

Most patients were treated with chloroquine, primaquine, mefloquine or quinine + doxycycline. In some cases, atovaquone/proguanil, artemether/lumefantrine, piperaquine + dihydroartesiminin or quinine + clindamycin were used for treatment. In cases of intravenous administration, a switch to oral administration was made after clinical improvement.

Treatment related adverse events were only described in one paper: The treatment of a 12-year-old Greek boy with chloroquine (10 mg/kg) and primaquine (0.5 mg/kg) led to the formation of methemoglobin (MetHb), despite the prior assessment of glucose-6-phosphate-dehydrogenase-level. This was discovered by performing an arterial blood gas analysis due to a reduced measured oxygen saturation on the third day of treatment. Treatment was initiated with ascorbic acid and daily determination of MetHb levels until the end of treatment. He also developed nausea and vomiting, which are known side effects of chloroquine [54].

No relapses were mentioned.

Regarding the occupation of autochthonous malaria cases: Employment was mentioned in 15 cases. These include working in agriculture (Greece 2x, Italy 4x, France 1x), a worker at a sewage plant (Germany 2x), as a schoolteacher (Greece), physician (Germany), a worker at a pig farm (Spain), a beach vendor (Spain), worker at an airport (France) and as a fuel supplier (France). In contrast to airport malaria, where the *Anopheles* mosquito is often imported as a stowaway mosquito, local breeding sites were sought in the region of occurrence of autochthonous or locally transmitted cases.

In entomological surveys the areas surrounding the residence or working place of the malaria cases was searched for breeding sites. Living next to a national park or working at a sewage plant or in agriculture gives some opportunities for stagnant water. If potential breeding sites were found, mosquito traps were set up and then analyzed after a few days. Mostly there were no *Anopheles* mosquitoes found and if they found, none of them was infected with *Plasmodia* parasites.

In 8 cases the distance to an airport was looked at as part of the entomological survey and origin search but was ruled out as a possible cause.

## 4 Discussion

Although rare, malaria is still a threat in Europe especially in the context of airport/seaport and autochthonous malaria. Changing climate with rising temperatures and increased precipitation may offer conducive conditions for the *Anopheles* mosquito to proliferate and to effectively transmit malaria especially *P. vivax* malaria. With increasing temperatures, the mosquitoes can establish themselves at higher altitude and latitude [2]. Humidity and at least 15 consecutive days at a temperature over 15 °C are needed for completing a development cycle of the *Plasmodium* parasite in the *Anopheles* mosquito [30]. Respectively, dependent on the *Plasmodium* species some 105 days with a temperature over 14.4 °C are needed for completion of the parasite development cycle in the mosquito and becoming potentially infectious [2]. Days and periods with such conditions are becoming more frequent during summertime in Europe.

With the constant increase in travel and migration, European *Anopheles* mosquitoes can become infected when they bite a returned traveler who is infected with *Plasmodium* parasites.

The importance of airport/seaport malaria is due not to a large number of cases but to the frequently delayed or missed diagnoses, particularly in cases presenting far away from the airport/port region where the malaria was acquired. The absence of a history of travel to a malaria-endemic area creates a lack of clinical suspicion, leads to a high case fatality rate and sometimes diagnosis is only made at autopsy. Rising temperatures in Europe and increased travel may facilitate the importation of competent *Anopheles* vectors, particularly *Anopheles gambiae* from Africa, some of which will be malaria infected.

Models using air traffic volume and seasonality may predict the risk of stow away mosquitoes on air routes arriving from malaria endemic areas and may be a basis for the implementation and timing of control measures such as aircraft disinsection [81]. Physicians, particularly those practicing in the vicinity of large airport hubs need to “*think malaria*” in unexplained febrile illnesses even if there is no history of travel to malaria endemic areas. In addition, the information and education of airport employees can also be taken into consideration for a certain period of time as was done in Frankfurt am Main in 2022 [15].

Initiated treatment with antibiotics can also contribute to a delay in diagnosis, as for example tetracycline, cotrimoxazole, erythromycin, clindamycin and quinolones [82] have some anti-malaria activity but are not sufficient to reduce parasitemia.

The importance of autochthonous malaria is thus related to delayed or missed diagnoses, particularly in cases where tropical diseases are not suspected as the history of travel to a malaria-endemic region is missing. The lower case fatality rate (CFR) with autochthonous malaria cases is most likely due to the fact that *P. vivax* is the predominant *Plasmodium* species transmitted and this is associated with a lower CFR.

In order to prevent sustained local transmission, anti-mosquito measures were taken by the authorities in some countries. For example, in Greece after the case cluster in 2011, the reported cases were traced, homes visited and face-to face interviews were done to elucidate the details of the cases [4]. Entomological surveys to identify mosquito breeding areas were also a part of this campaign. Careful risk communication is required and selective testing programs are needed with the involvement of the key health facilities [57]. Gentilini and Danis (1981) pointed out that the focus in Europe has now switched to combat against *Culex* and *Aedes* mosquitoes instead of *Anopheles* mosquitoes and that this may open the door for the potential reintroduction of malaria in some areas in Europe [83].

This systematic review had some limitations: The variation of the content in between the publications is wide – from minimal information with year of occurrence, age, type of malaria and survival to very detailed investigations including genomic analyses and epidemiological investigations. We were also dependent on the digitalization of older cases, as the search was conducted in online databases.

## 5 Conclusions

Even though malaria is considered to be eliminated in Europe, rare cases of airport/seaport and autochthonous malaria will continue to occur and are associated with a high case fatality rate. A changing climate with rising temperatures and increased precipitation may facilitate the establishment and proliferation of competent *Anopheles* vectors. Increased human migration and travel with malaria parasite carriage may contribute to the reemergence of autochthonous malaria in Europe. Health care professionals and persons working at airports or living close by need to be sensitized to the risk of airport malaria.

## CRediT authorship contribution statement

**Daniela Balzli:** Writing – original draft, Visualization, Investigation, Formal analysis. **Nejla Gültekin:** Writing – review & editing. **Zeno Stanga:** Writing – review & editing. **Ismail Ülgür:** Writing – review & editing. **Nadja Hedrich:** Writing – review & editing. **Jan Fehr:** Writing – review & editing. **Patricia Schlagenhauf:** Writing – review & editing, Writing – original draft, Supervision, Conceptualization.

## Funding

This research did not receive any specific grant.

## Declaration of competing interest

The authors declare that they have no known competing financial interests or personal relationships that could have appeared to influence the work reported in this paper.
